# Bridging Basic and Clinical Research in Early Life Adversity, DNA Methylation, and Major Depressive Disorder

**DOI:** 10.3389/fgene.2019.00229

**Published:** 2019-03-22

**Authors:** Amanda Brown, Laura M. Fiori, Gustavo Turecki

**Affiliations:** McGill Group for Suicide Studies, Department of Psychiatry, McGill University, Montreal, QC, Canada

**Keywords:** epigenetics, major depressive disorder, early life adversity, DNA methylation, biomarkers, antidepressant therapies

## Abstract

Early life adversity (ELA)- including childhood physical, emotional, and sexual abuse, as well as childhood neglect- is an important predictive factor for negative psychopathology, including Major Depressive Disorder (MDD). ELA can epigenetically regulate key emotional and behavioral systems in ways that can stably persist into adulthood and contribute to the development of MDD and other psychopathology. DNA methylation has been one of the most investigated forms of epigenetic regulation in ELA to MDD pathway. From these studies, genes and sites associated with ELA/MDD have been identified and should be further investigated in order to identify potential avenues for intervention.

## Introduction

Early life adversity (ELA)- which includes forms of child maltreatment such as physical abuse, sexual abuse, psychological and emotional abuse, and childhood neglect- remains a major public health and welfare issue in high income countries (Krugers et al., 2017). It is defined as any act that is either actively conducted, or neglected to be conducted, by a parent or caregiver that either harms or has potential to harm a child, regardless of intent. These acts include physical abuse such as hitting, punching, beating, strangling, and shaking, sexual abuse such as penetration, sexual contact, and exposure to sexual activity, emotional and psychological abuse such as terrorizing, intimidating, and belittling, and forms of neglect such as failure to provide clothing, food, shelter, or neglect in seeking medical care for a child (Leeb et al., 2008). Overall, the various forms of ELA are estimated to affect approximately 10–15% of children (typically categorized as individuals under the age of 18). Specifically, between 4 and 16% of children experience physical abuse, and approximately 15% of children are subjected to sexual abuse annually (Fergusson et al., 2000; Woodman et al., 2008; Gilbert et al., 2009).

In the United States, approximately 80% of substantiated ELA cases are found to be perpetuated by either one or both parents, with 77% of physical abuse, 26% of sexual abuse, 81% of emotional/psychological abuse, and 87% of neglect cases being perpetrated by a parent (US Department of Health and Human Services, 2008; Jonson-Reid et al., 2012; Nemeroff, 2016). Additionally, 29% of sexual abuse cases are perpetrated by a non-parent relative (Fergusson et al., 2000). It is worth noting that these estimates reflect only officially reported and substantiated cases, and that the actual burden of ELA likely exceeds these estimates.

This substantial phenomenon of ELA is of particular importance as a public health issue considering that repeated exposure to ELA, as well as exposure to multiple types of ELA, has the potential to inflict severe and lasting physical and psychological consequences that represent serious public healthcare and legal costs, as well as increased risk of mortality (Gilbert et al., 2009). ELA has been shown to contribute significantly to the risk of criminal behavior, childhood/adolescent behavioral problems, general physical heath, obesity, promiscuity, prostitution and sex trading, substance use, attempted suicide, and various psychopathologies such as post-traumatic stress disorder (PTSD), and Major Depressive Disorder (MDD), bipolar disorder, and conduct disorder (Thomas et al., 2008; Gilbert et al., 2009; Weder et al., 2014; Nemeroff, 2016; Krugers et al., 2017).

The role of ELA in the development of MDD is of particular interest with regard to public health and welfare, as MDD is the leading cause of global disability, affecting over 300 million people worldwide (World Health Organization [WHO], 2017). It is characterized by persistent depressed mood and loss of interest and pleasure, and may also include symptoms such as weight loss or gain, insomnia or hypersomnia, fatigue, loss of motivation or concentration, recurring thoughts of death, and suicidal ideation (Zajecka et al., 2013).

It is believed that exposure to repeated ELA may increase risk for MDD by cumulating in a hostile and unstable early environment that may trigger adaptive responses in the brain in crucial response systems (such as stress and emotional regulation). There is evidence that these regulatory changes can stably and radically impact aspects of personality development and cognitive functioning in ways that can increase the risk for MDD and associated psychopathologies (Saavedra et al., 2016). The etiology underlying the pathophysiology of MDD is a complex and often varied interplay between genetic, epigenetic, environmental, clinical, and social factors. Research suggests that one of these factors-ELA-can trigger epigenetic alterations (through events such as DNA methylation) in neural systems and genes associated with increased stress response [such as the in the hypothalamic pituitary adrenal axis (HPA)] as a survival mechanism. The persistence of these epigenetic changes often become maladaptive as the early life environment changes, but the altered function of the stress systems does not. While it is not fully understood how these alterations contribute specifically to MDD symptomology, DNA methylation events in these systems in individuals who have experienced ELA have been associated with MDD (Saavedra et al., 2016; Pishva et al., 2017).

It is worth noting that ELA itself is a complex set of phenomena that increases the complexity of MDD pathophysiology, and includes different severities and consequences. There is evidence of a dose-response relationship between ELA and psychopathologies such as MDD, wherein a higher severity of childhood adversity has been linked to increased incidence of psychopathology, higher comorbidity among psychiatric diagnoses, and more severe symptoms within diagnoses (Edwards et al., 2003; Chapman et al., 2004; Heim and Binder, 2012).

With respect to MDD specifically, ELA has been shown to correspond to a more severe course of depression, particularly in terms of increased chronicity, resistance to psychopharmacology and psychotherapy, and greater presentation of atypical symptoms (such as hypersomnia, increased appetite, and increased sensitivity to rejection) (Withers et al., 2013).

There is evidence that certain factors, such as microstructural differences in white matter, as measured by fractional anisotropy, and increased neural fiber connectivity, may be protective against the development of MDD in individuals who experienced ELA. Fractional anisotropy measures the anisotropic movement of water molecules (thought to be important in extrasynaptic communication) associated with fiber bundles (Morgan et al., 2013). One study found that unaffected first degree relatives of individuals with MDD, who also experienced ELA, had significantly increase fractional anisotropy in the right frontal and orbitofrontal lobes, corpus callosum, inferior fronto-occipital fasciculus (IFO), left superior longitudinal fasciculus (SLF) and right fornix (Frodl et al., 2012).

However, resilience is a complex topic in ELA and MDD research, and personal coping mechanisms, behavioral factors, and the presence of positive interpersonal influences concurrent with the ELA were not examined in this study. Epigenetic regulation related to the growth factor brain-derived neurotrophic factor (BDNF) may also play a role in conferring resilience to MDD in these circumstances, and deserves further investigation (Krishnan et al., 2007). Due to all of these intersecting factors, resilience from MDD in the presence of ELA is therefore difficult to study and adequately quantify. Regardless, while certain biological and environmental factors may act as protective factors against MDD development in individuals who have experienced ELA in ways that are not yet fully understood, it remains true that ELA is one of the greatest risk factors for the development of MDD (Saavedra et al., 2016).

Considering the prominent role that severe ELA plays in the development of MDD, and the overall prevalence of MDD, these figures indicate that ELA imposes a substantial social, economic, and public health cost upon society. Due to this, research identifying the mechanisms of action in the ELA to MDD pathway could prove to be a great asset in the development of clinical treatments of MDD. One mechanism by which ELA has been shown to influence emotional and stress regulation systems is epigenetic regulation, such as DNA methylation (Cecil et al., 2016). Although other forms of ELA have been examined with respect to the ELA to MDD pathway, DNA methylation remains one of the most predominately studied, and will serve as the focus of this review. Here, we will first explore how DNA methylation has been implicated in ELA and MDD, and then examine how DNA methylation also has the potential to impact clinical research into MDD diagnosis and treatment.

## DNA Methylation in ELA/MDD

Epigenetic regulation describes any regulatory event which causes alterations in gene expression without changes to the original DNA sequence. Various forms of epigenetic regulation have been extensively studied as causal factors in the depression endophenotype in individuals who have experienced ELA, including DNA methylation, histone modifications, and microRNA.

DNA methylation is one of the most well-investigated forms of epigenetic regulation and has been studied as a causal factor in the development of MDD in both candidate gene and genome wide studies (Lutz et al., 2015). During DNA methylation, the DNA is covalently modified via addition of a methyl group to a cytosine nucleotide. This phenomenon is particularly common in cytosine-guanine dinucleotides (CpGs) (Jin et al., 2011). The methylation status of the nucleotide is read by methyl CpG-binding domain (MBD) proteins- such as MeCP2 and MBD1-4- which interact with histone deactylases and DNA-methyltransferases that induce chromatin condensation (Chen et al., 2003; Chahrour et al., 2008; Guy et al., 2011). When methylation is found in promoter regions, it can interfere with transcription factor binding and lead to gene silencing. Conversely, site specific dissociation of the MBDs – specifically MeCP2- have been associated with demethylation, and have been implicated to play a major role in maintaining DNA methylation status (Martinowich et al., 2003).

During prenatal development, DNA methylation patterns are crucial to the process of cell differentiation. However, changes in DNA methylation occur beyond this period, as a mechanism that helps the genome adapt to external signals in the environment. This form of DNA methylation allows the genome to adjust its function through diverse -yet stable- changes. This is one possible mechanism by which environmental stressors such as ELA may trigger epigenetic changes that could ultimately contribute to psychopathology such as MDD (Turecki, 2014).

## Genome-Wide and Multiple Loci Studies

### Human Post Mortem Brain Tissue Studies

In addition to approaches based on the investigation of candidate genes, the role of DNA Methylation in ELA/MDD has also been examined using larger-scale, genome-wide studies. One of the earliest studies examined hippocampal tissue from French Canadian men with a history of severe childhood abuse (who also died by suicide) using meDIP, an antibody targeting methylated cytosines, coupled with an tiled array containing gene promoters. Differential DNA methylation indicated that 248 sites were hypermethylated and 114 were hypomethylated (Labonté et al., 2012). Significant differences involved genes implicated in cellular or neural plasticity, and some of these findings included histone cluster 2 H2ab (*HIST2H2AB*), nuclear receptor subfamily 1, group D, member 1 (*NR1D1*); and amyotrophic lateral sclerosis 2 (*ALS2*). Specifically, *in vitro* analysis of *ALS2* methylation levels indicated functional effects on gene expression- supporting the hypothesis that ELA contributes to cell-type specific reprogramming of the epigenome in ways that may influence emotional and behavioral dysregulation (Lutz et al., 2015).

In another region of the brain that is hypothesized to be implicated in ELA/MDD- the anterior cingulate cortex (ACC) – genome-wide DNA methylation was assessed using reduced representation bisulfite sequencing (RRBS) in human post mortem brain tissue of depressed suicides who experienced ELA (Table 1) (Lutz et al., 2017b). In this study, the most significantly differentially methylated genes were those related to myelin and oligodendrocytes. Specifically, the three most differentially methylated genes were *LINGO3* (which codes for LINGO proteins that are implicated in myelination), *POU3F1* (a transcription factor that controls myelination), and *ITGB1* (an integrin that mediates oligodendrocyte and axonal interactions). These findings suggested that ELA may lead to myelin alterations, a conclusion also supported by both transcriptomic and morphological changes observed in this study using other techniques (Table 1) (Lutz et al., 2017b), and consistent with pre-clinical studies indicating that a critical period in the early social environment regulates myelination that in turn is essential for normal cognitive function (Makinodan et al., 2012).

**Table 1:** Summary of studies investigating clinical applications of ELA and MDD research involving- or related to – DNA methylation in ELA and MDD.

Reference	Sample Size^∗^	Adversity Group	Sample	Purpose	Methodology	Data
Fuchikami et al., 2011	38 (20+18)	MDD	Blood	Diagnostic biomarker	Bisulphite sequencing	DNA methylation in BDNF
Lopez et al., 2013	25	Treatment naïve MDD	Blood	Antidepressant efficacy biomarker	qRT-PCR	BDNF concentration
Perroud et al., 2013	167 (52+115)	BPD and ELA	Blood	Psychotherpay efficacy biomarker	High-resolution melt assay	DNA methylation in BDNF
Mundorf et al., 2018	60	MDD and ELA	Buccal cells	Diagnostic biomarker	Bisulfite sequencing (with EpiTect Kit)	DNA methylation in MORC1
Covington et al., 2009	Mouse model	Chronic social defeat	Mouse NaC tissue	Antidepressant efficacy biomarker	Gene expression arrays	Expression levels of 12 genes in the NAc
Melas et al., 2012	Mouse model	Flinders sensitive line (FSL) genetic rodent model of depression	Mouse prefrontal cortex tissue	Antidepressant efficacy biomarker	Pyrosequencing	DNA methylation in P11
Lutz et al., 2017b	78 (52+26)	MDD/suicide +ELA and MDD/suicide without ELA	Human post mortem ACC Tissue	Quantifying myelination alterations in MDD	bisulfite sequencing/RNA sequencing/stereology and coherent anti-Stokes Raman scattering microscopy.	Methylation and expressionlevels of myelin related genes, and imaging of oligodendrocytes and myelinated axons
Kaufman et al., 2018	157	Children who experienced ELA	Saliva and neuroimaging data	Predicting depression in children who experienced ELA	Illumina 450?K beadchip/fMRI	DNA methylation in OTX2 and functional connectivity between the ventromedial prefrontal cortex and structures of the medial frontal cortex
Sacchet and Gotlib, 2017	80 (40 +40)	MDD	Neuroimaging data	Quantifying myleination alterations in MDD	qMRI	Whole brain, Nac, lateral PFC myelin levels


*^∗^sample size: total (adversity group+ control cohort)*.

### Peripheral Tissue Studies

Other studies evaluated DNA methylation changes associated with ELA, using peripheral samples. A study investigating methylation in the promoter region of over 20 000 genes, including 489 coding for miRNAs, in the blood DNA of males in the 1958 British Birth Cohort found 997 differentially methylated gene promoters associated with ELA (311 hypermethylated and 686 hypomethylated), which were enriched for genes involved in processes such as transcriptional regulation and development. Additionally, they observed ELA associated methylation in thirty-one miRNA genes, six of which showed hypermethylation consistent with hypomethylation observed in their downstream gene targets (Suderman et al., 2015). Another genome wide study by Weder et al. (2014) used the Illumina 450K BeadChip array to determine potential sites of differential methylation that could predict depression in saliva samples collected from maltreated children compared to non-traumatized. Three genes emerged as predictors of depression in combination with ELA: DNA-Binding Protein Inhibitor ID–3 (*ID3*); and Tubulin Polymerization Promoting Protein (*TPPP*) (Weder et al., 2014); and the neurotransmitter gene glutamate receptor, ionotropic *N*-methyl-D-aspartate 1 (GRIN1) (Weder et al., 2014).

More recently, the Illumina 450K BeadChip array was used by the same group in another study of ELA and MDD that sought to predict differential DNA methylation in maltreated children. In this study (Kaufman et al., 2018), methylation of the Orthodenticle homeobox 2 (*OTX2*) gene significantly predicted depression in saliva samples of maltreated children compared to controls (Table 1) (Kaufman et al., 2018).

### Neuroimaging

Imaging data revealed that methylation of *OTX2* is associated with increased functional connectivity between the ventromedial prefrontal cortex and structures of the medial frontal cortex that have been implicated in MDD- such as the paracingulate gyrus, frontal pole, and subcallosal gyrus (Table 1) (Kaufman et al., 2018).

## Candidate Genes

### HPA Axis

Numerous candidate gene studies have also investigated the relationship between ELA, depressive symptoms, and DNA methylation. The majority of these studies have been performed in peripheral samples such as blood and saliva. However, studies utilizing brain and CNS related tissues and systems have also been widely performed in both animal models and human post mortem tissue studies. One biological system in particular, the hypothalamic-pituitary-adrenal (HPA) axis has been the focus of many of these studies.

The HPA axis is a primary stress response system in mammals that has been extensively studied for its role in various psychopathologies including MDD, bipolar disorder, hypersexual disorder, and behaviors like habitual smoking (Rohleder and Kirschbaum, 2006; Chatzittofis et al., 2016; Murri et al., 2016). The HPA response begins in neurons in the paraventricular nucleus (PVN) of the hypothalamus, where the neurohormones CRF and arginine vasopressin (AVP) are released into blood vessels between the hypothalamus and pituitary gland. The pituitary gland is then stimulated to secrete adrenocorticotropic hormone (ACTH) into the circulation to induce the synthesis and secretion of glucocorticoids like cortisol from the adrenal glands (Pariante and Lightman, 2008; Perroud et al., 2011; Keller et al., 2016).

Studies have demonstrated that ELA can foster HPA axis dysfunction in ways that increase susceptibility to depression. Specifically, this dysfunction may result from disturbances to the negative feedback system of the HPA axis, which is partially controlled by the expression of the glucocorticoid receptor (*GR/NR3C1*) in the hippocampus, expression of the *CRF* gene, or changes in *FKBP5* expression- a gene responsible for stabilizing the conformation of the GR (Pariante and Lightman, 2008; Keller et al., 2016).

### Animal Models

Animal models of maternal care have provided a substantial contribution to the understanding of the relationship between the early environment and functioning of the HPA axis. Rats who receive decreased maternal attention in the form of low licking-grooming (LG) behavior in the first week of life have been found to show increased HPA stress responses and hypothalamic corticotrophin releasing factor (CRF) expression, and decreased GR expression and glucocorticoid feedback sensitivity compared to high LG rats. This is due in large part to differential methylation of the *GR/NR3C1* gene which is downregulated in low LG rats, with increased DNA methylation being observed in the promoter region (exon 17) of *NR3C1* in the hippocampus, which persists into adulthood (Weaver et al., 2007, 2014).

In addition to *NR3C1*, hypothalamic corticotrophin releasing factor (CRF), also plays a key role in this feedback system and exerts control over the HPA axis and other stress responses in the brain. Mice subjected to chronic social defeat display decreased DNA methylation in the *CRF* gene, leading to sustained upregulation of *CRF* in neurons of the paraventricular nucleus (PVN) in the hypothalamus (Elliott et al., 2010). This ELA model leads to stress-induced phosphorylation of MeCP2, which results in MeCP2 being dissociated from the inhibitory complex of methylated CpG sites in the *CRF* promotor region, resulting in increased transcription. This mechanism of *CRF* upregulation is supported by the observation that mice with MeCP2 knockouts in the PVN present with abnormal physiological stress responses (Fyffe et al., 2008). Conversely, a rat model of chronic mild stress actually demonstrated increased DNA methylation of the *CRF* promoter in the PVN, indicating that sex-specific and/or dose-dependent effects may play a role in altered HPA axis activity (Sterrenburg et al., 2011).

### Human Post Mortem Brain Tissue Studies

Human post mortem brain tissue studies have also demonstrated ELA-associated epigenetic changes in the HPA axis, as hippocampal tissue obtained from individuals who died by suicide and experienced ELA (particularly severe childhood abuse) displayed an increase in *NR3C1* methylation, and decreased *NR3C1* expression (McGowan et al., 2009; Suderman et al., 2012).

Finally, in the neurons of the hypothalamic paraventricular nucleus, increased arginine vasopressin (AVP) release accompanied by HPA axis hypersensitivity has also been linked to hypomethylation in reduced maternal care animals, in a similar mechanism to *CRF* hypomethylation (Murgatroyd et al., 2009).

### Peripheral Tissue Studies

The GR regulating gene, *FKBP5*, has also been the subject of several studies investigating differential methylation in peripheral tissue. Certain polymorphisms of *FKBP5* (rs1360780, rs9296158, rs3800373, and rs9470080) interact with ELA to predict MDD and suicide attempts (Roy et al., 2010; Appel et al., 2011). One study by Klengel et al. (2012) examined *FKBP5* methylation levels in the blood of individuals with a history of ELA and found hypomethylation in intron seven. Another study collected saliva samples from children who had experienced ELA, and assessed them for DNA methylation levels at two CpG sites in intron seven of *FKBP5* (Tyrka et al., 2015). Once again, hypomethylation was found at both sites in intron seven. It was hypothesized that increased cortisol release following ELA in *FKBP5* risk allele carriers would signal differential methylation in *FKBP5* and disrupt the feedback loop responsible for *FKBP5* and GR activity, leading to stress response dysregulation and increased susceptibility to MDD (Tyrka et al., 2015).

## Neurotransmitters

DNA methylation in neurotransmitter genes has also been explored in CNS and peripheral human samples, as well as animal studies, with respect to the ELA to MDD pathway. In addition to the previous discussed Weder et al. study that implicated the neurotransmitter gene glutamate receptor, ionotropic *N*-methyl-D-aspartate 1 (GRIN1) in ELA/MDD, one gene, *5-HT2A*, has been explored in the prefrontal cortex (PFC) for its potential contribution to ELA related MDD and suicide (Du et al., 2000, 2001; De Luca et al., 2007, 2009). One particular *5-HT2A* polymorphism, the C allele (C102T), has been associated with depression and suicide, and has been found to be more abundant in the PFC of suicides. Although non-significant hypomethylation differences were found in the C allele in suicides, hypermethylation was reported in suicide ideators, indicating potential differential methylation patterns between these two groups (De Luca et al., 2009).

In the anterior insula, downregulation of the kappa opioid receptor (*Kappa*) and decreased DNA methylation in the second intron of the *Kappa* gene has been found in depressed suicides who experienced ELA. This intron serves as a genomic enhancer in GR binding regulated *Kappa* expression; indicating a mechanism by which endogenous opioids act on stress systems (Lutz et al., 2017a).

Numerous studies have investigated the serotonin transporter gene *SLC6A4*, which facilitates neurotransmitter reuptake at serotonergic synapses. Hypermethylation has been demonstrated at various CpG sites in ELA cohorts depending on sex, ELA magnitude, and type of ELA experienced (ex. physical versus sexual abuse) (Vijayendran et al., 2012; Alexander et al., 2014; Booij et al., 2015; Frodl et al., 2015). ELA related *SLC6A4* promoter DNA methylation status was also found to be significantly associated with more severe pre-treatment presentation of depressive symptoms, elevated stress, and higher family history of psychopathology (Menke et al., 2012).

## Growth Factors

Growth factors, such as brain derived neurotrophic factor (BDNF) and glial cell-derived neurotrophic factor (GDNF), also show differences in expression and DNA methylation related to ELA and MDD. In particular, BDNF has been the subject of significant basic and clinical research with regards to ELA/MDD, as it has been the subject of candidate gene studies, and shows promise as a biomarker and target for epigenome targeted antidepressant therapy.

In the nucleus accumbens (NAc), glial cell-derived neurotrophic factor (*GDNF)* expression appears to be reduced in chronic early life stressed mice with anxiety- and depressive-like behaviors. This reduction appears to be due to increased DNA methylation related to increased MeCP2 binding (Chahrour et al., 2008; Uchida et al., 2011).

The brain derived neurotrophic factor (*BDNF*) gene is expressed in the adult PFC, and plays a critical role in neural and behavioral plasticity, and the development of psychiatric disorders such as depression, bipolar disorder, and schizophrenia when coupled with ELA (Kundakovic et al., 2014). Hypomethylation of *BDNF* has been observed in both human PFC tissue in individuals who have experienced ELA, as well as in the PFC of rats exposed to early life stress (Roth et al., 2009; Roth and Sweatt, 2011).

With a significant amount of research substantiating the biological relevance of genes such as *BDNF* in the development of ELA related MDD, reliable estimates of methylation at this locus could potentially be used as a diagnostic tool (Table 1) (Fuchikami et al., 2011; Kundakovic et al., 2014).

Taken in context with the established ELA related behavioral vulnerabilities that *BDNF* methylation alterations represent, blood *BDNF* expression and methylation levels could potentially be of clinical use as a tool in clinical care of psychopathologies such as MDD, when coupled with the presentation of symptoms and a reported history of ELA (Shonkoff et al., 2009).

One study examined two *BDNF* CpG islands (I and IV) as potential diagnostic biomarkers of MDD. Examining methylation levels at CpG islands I and IV in human cohorts of MDD patients and healthy controls, it was found that CpG 1, but not IV, levels could accurately discriminate controls from the MDD cohort. While this study utilized a relatively small sample size (20 MDD vs. 18 control individuals), it indicated that DNA methylation levels have the potential to be a valuable resource in clinical diagnosis as a biomarker (Table 1) (Fuchikami et al., 2011).

Clinical research focused on the epigenetic effects of ELA has the potential to not only create new diagnostic procedures, but also to create novel antidepressant therapies that target the epigenome or use the epigenome as a means to evaluate antidepressant efficacy (Qiu et al., 2017). BDNF in particular appears to be influenced by antidepressant treatment (Fuchikami et al., 2016). Notably, in several preclinical animal models, treatment with the histone deacetylase (HDAC) inhibitor sodium butyrate has yielded reduced depressive and manic-like behaviors, as well as reversed decreased expression in BDNF and other neurotrophic factors in chronically stressed animal models (Moretti et al., 2011; Resende et al., 2013). Similarly, animal models treated with the antidepressant imipramine present with reversed behavioral consequences of early life stress in the *BDNF* promoter region (Tsankova et al., 2006; Hollis et al., 2010, 2011; Covington et al., 2011).

It has been suggested that effective antidepressant treatment should correspond to an increase in peripheral *BDNF*, and that a sustained lack of increase in *BDNF* over the first week of treatment likely predicts treatment resistance (Tadić et al., 2011). This finding is based on *BDNF* exon IV promoter methylation data from an MDD cohort being treated with several antidepressants. This effect was mirrored by findings showing that citalopram treatment in MDD patients increased *BDNF* expression in treatment responders, and significantly reduced H3K27me3 levels at the *BDNF* IV promoter (Table 1) (Lopez et al., 2013).

## Animal Models of OTX2

In an elegant study, Peña et al. (2017) described that juvenile knockdown of the transcription factor orthodenticle homeobox 2 (*Otx2)* in the ventral tegmental area (VTA) – a brain reward region- increases stress susceptibility similarly to early life stress. This knockdown of *Otx2* was also associated with downregulation of several target genes, many of which play a role in brain development. Animals in this model also exhibited depressive-like behaviors following a second exposure to stress in adulthood. Based on these findings, the researchers proposed that a “two-hit” stress model may be in effect, wherein ELA increases stress susceptibility in the VTA via *Otx2* mediation, contributing to a depression-like state and sustained transcriptional alterations in adults following adult social defeat. As the association between ELA/MDD and OTX2 has been also reported in humans (Kaufman et al., 2018), continued investigation into how *Otx2* mediates sustained stress susceptibility is warranted.

## Structural Studies of Myelination

The role of myelination in ELA/MDD may also be of potential clinical interest. Myelination is essential in developing and maintaining complex cognitive and behavioral functions, as it increases action potential transmission per unit time, thereby increasing the connectivity and information processing capacity of the human brain (Grydeland et al., 2013). Myelination occurs when oligodendrocytes cells generate myelin, which insulates the neuronal axons and facilitates electrical signal propagation, helping maintain the circuitry of neural networks (such as the axon segments within the cortex), which are crucial to optimizing basic cognitive and behavioral functions (Haroutunian et al., 2014; Long and Corfas, 2014). Due to this, reductions in cortical oligodendrocytes and deficits in myelin gene expression have been associated with cognitive and behavioral dysregulations which include- but are not limited to- schizophrenia, bipolar disorder, and MDD (Uranova et al., 2004). Intracortical myelin abnormalities vary across psychopathologies, with deficits in white matter volume and myelination being more prominent in bipolar disorder and MDD (Mosebach et al., 2013). With respect to MDD specifically, quantitative magnetic resonance imaging (qMRI) analysis has revealed reduced myelin levels in the white matter of brain regions such as the nucleus accumbens (NAcc) and lateral prefrontal cortex (LPFC) (Table 1) (Sacchet and Gotlib, 2017).

Structural alterations in white matter have also been associated with ELA in MRI studies, with more recent MRI strategies being able to more effectively investigate white matter myelin content to the level of bundles with hundreds or thousands of axons (Stikov et al., 2015). Although oligodendrocytes and myelin were long thought to be static, these imaging studies, as well as animal studies, have demonstrated that various forms of learning, as well as social and environmental conditions, correlate with structural changes to white matter. This suggests an important role for myelination in brain plasticity (Long and Corfas, 2014).

In the previously summarized recent study by Lutz et al. (2017b), state of the art microscopy methods were utilized to more precisely measure microstructural changes to white matter myelin that are associated with ELA. They discovered that in post mortem brain tissue from adults who experienced ELA and died by suicide, axonal integrity and myelination of individual fibers was disrupted, particularly in small-diameter axons which may be related to cortico-cortical projections. These structural alterations were found in conjunction with cell type-specific differences in DNA methylation of oligodendrocyte genes such as *POU3F1* and *LINGO3* in oligodendrocytes (but not neurons), and global decreased expression of a large collection of myelin related genes in the cingulate cortex. These results suggest that ELA may contribute to a neurobiological vulnerability that increases the risk of psychopathology throughout life in affected individuals (Table 1) (Lutz et al., 2017b). These findings may prove of future clinical significance, although further research into the brain region-specific effects of ELA/MDD on myelin is needed before any potential antidepressant therapies or diagnostic procedures related to myelination can be explored further.

## Additional DNA Methylation Studies of Clinical Interest

BDNF is not the only gene that has potential as a biomarker for MDD in individuals who have experienced ELA. The MORC family CW-type zinc finger 1 (*MORC1)* gene, has been found to be hypermethylated in buccal cells of individuals who experienced ELA and scored high on the Beck Depression Inventory. Researchers have suggested that *MORC1* could reliably be used as a non-invasive diagnostic biomarker for MDD in individuals who have been affected by ELA (Table 1) (Mundorf et al., 2018).

Other genes implicated in social defeat models of ELA may also serve as promising targets for the future development of novel antidepressant treatments. One mouse model of ELA that presented with altered gene expression in 12 genes in the NAc, including genes coding for actin cytoskeleton reorganization machinery, transcription factors, signaling molecules, and neurotransmitter receptors, that was reversed by treatment with the antidepressant MS-275 (Table 1) (Covington et al., 2009).

In the prefrontal cortex, DNA methylation of the P11 promoter was reduced in MDD patients via administration of escitalopram, increasing P11 and decreasing the presence of DNA methyltransferases (Table 1) (Melas et al., 2012). The epigenetic changes detailed in these studies represent both potential targets for antidepressant therapy, as well as potential biomarkers of drug efficacy.

While there has been an ample amount of basic research on the ELA to MDD pipeline examining regions and genes that are differentially methylated in ELA/MDD, clinical research expanding on these findings has been less bountiful. Further clinical studies that examine expression levels of key genes in peripheral tissue (such as buccal swabs or blood) before, during, and after antidepressant treatment in ELA/MDD subjects could feasibly be undertaken to help evaluate the efficacy of many antidepressant treatments, whether they be pharmacological, psychotherapeutic, or through methods such as brain stimulation- the last two of which (to the authors’ knowledge) – remain to be explored.

## Conclusion

The studies described herein have contributed to our understanding of how ELA can epigenetically regulate behavioral and emotional response systems in the brain in ways that contribute to- and increase the vulnerability to- psychopathologies such as MDD. The research indicates that genes that code for the HPA axis, neurotransmitters, growth factors such as BDNF, transcription factors such as OTX2, and myelination/oligodendrocytes may all play a role in this pathway, demonstrating how diverse and complex the epigenetic alterations associated with ELA likely are. Due to this complexity, it is perhaps not surprising that clinical progress-such as the development of biomarkers for ELA/MD and antidepressant therapies that target the epigenome-has thus far been limited and preliminary. This is why further studies will be necessary before the full potential clinical significance of the basic research into the ELA/MDD pathway can be determined.

## Author Contributions

AB wrote most of the manuscript, including the entire first draft. LF carried out substantial editing, revisions, and rewrites. GT edited and revised the final draft.

## Conflict of Interest Statement

The authors declare that the research was conducted in the absence of any commercial or financial relationships that could be construed as a potential conflict of interest.
